# Cerebellar Non-Invasive Brain Stimulation: A Frontier in Chronic Pain Therapy

**DOI:** 10.3390/jpm14070675

**Published:** 2024-06-23

**Authors:** Valerio Sveva, Alessandro Cruciani, Marco Mancuso, Francesca Santoro, Anna Latorre, Marco Monticone, Lorenzo Rocchi

**Affiliations:** 1 Department of Anatomical and Histological Sciences, Legal Medicine and Orthopedics, University of Rome “Sapienza”, Piazzale Aldo Moro 5, 00185 Rome, Italy; valerio.sveva@uniroma1.it; 2 Unit of Neurology, Neurophysiology, Neurobiology and Psychiatry, Department of Medicine and Surgery, Università Campus Bio-Medico di Roma, Via Alvaro del Portillo 21, 00128 Rome, Italy; alessandro.cruciani@unicampus.it (A.C.); francesca.santoro@unicampus.it (F.S.); 3 Department of Medicine and Surgery, Fondazione Policlinico Universitario Campus Bio-Medico, Via Alvaro del Portillo 200, 00128 Rome, Italy; 4 Department of Human Neuroscience, University of Rome “Sapienza”, Viale dell’Università 30, 00185 Rome, Italy; marco.mancuso@uniroma1.it; 5 Department of Clinical and Movement Neurosciences, UCL Queen Square Institute of Neurology, University College London, London WC1N 3BG, UK; a.latorre@ucl.ac.uk; 6 Department of Surgical Sciences, University of Cagliari, 09124 Cagliari, Italy; marco.monticone@unica.it; 7 Department of Medical Sciences and Public Health, University of Cagliari, 09124 Cagliari, Italy

**Keywords:** cerebellum, pain, neurophysiology, brain stimulation, transcranial magnetic stimulation, transcranial direct current stimulation, transcranial alternate current stimulation, electroencephalography, evoked potentials

## Abstract

Chronic pain poses a widespread and distressing challenge; it can be resistant to conventional therapies, often having significant side effects. Non-invasive brain stimulation (NIBS) techniques offer promising avenues for the safe and swift modulation of brain excitability. NIBS approaches for chronic pain management targeting the primary motor area have yielded variable outcomes. Recently, the cerebellum has emerged as a pivotal hub in human pain processing; however, the clinical application of cerebellar NIBS in chronic pain treatment remains limited. This review delineates the cerebellum’s role in pain modulation, recent advancements in NIBS for cerebellar activity modulation, and novel biomarkers for assessing cerebellar function in humans. Despite notable progress in NIBS techniques and cerebellar activity assessment, studies targeting cerebellar NIBS for chronic pain treatment are limited in number. Nevertheless, positive outcomes in pain alleviation have been reported with cerebellar anodal transcranial direct current stimulation. Our review underscores the potential for further integration between cerebellar NIBS and non-invasive assessments of cerebellar function to advance chronic pain treatment strategies.

## 1. Introduction

Chronic pain is a common and distressing problem affecting millions of people worldwide. Its prevalence is estimated to be around 30% to 50% of the adult population, and it is considered an important cause of disability and disease burden [1]; not only does it impact on mental health, social interactions, and quality of life, but it also impacts on the economy, increasing healthcare costs and productivity [2]. According to the International Association for the Study of Pain, chronic pain is defined as an unpleasant sensory and emotional experience associated with, or resembling that associated with, actual or potential tissue damage persisting for more than three months [3,4]. Pain is a consequence of biological, psychological, and social factors; hence, current guidelines recommend interdisciplinary treatment. A multimodal approach should include self-care, a healthy lifestyle, medical treatments such as opioid and non-opioid drugs, psychological therapies, and integrative treatments [2]. However, current therapies often fail to provide comprehensive relief and/or cause unbearable side effects. Non-pharmacological approaches such as physical therapy, acupuncture, and cognitive behavioural therapy represent an alternative or add-on therapy to standard treatments but may not adequately address mechanisms underlying chronic pain.

In view of the unsatisfactory outcomes of these approaches, neurostimulation offers additional aid to improve both the short- and long-term management of patients with chronic pain. Neuromodulation encompasses both non-invasive and invasive techniques, targeting central and peripheral nervous structures. Despite operating via diverse mechanisms, these therapeutic modalities exhibit convergence by promoting functional modifications (e.g., modulation of ion channels and neurotransmitter release) and facilitating neuroplasticity [5]. Prominent among non-invasive techniques are transcranial direct current stimulation (tDCS), repetitive transcranial magnetic stimulation (rTMS), and vagus nerve stimulation. In the realm of invasive stimulation techniques, dorsal root ganglion stimulation, epidural motor cortex stimulation, spinal cord stimulation, and deep brain stimulation are currently used.

To date, most positive results from non-invasive techniques come from stimulation of the primary motor cortex (M1), which produces analgesic effects by acting on various neural pathways and neurotransmitter systems [6]; the left dorsolateral prefrontal cortex (DLPFC) can be considered an alternative treatment location for patients with diffuse pain or severe comorbid depression. Similarly, the FDA has approved two handheld TMS devices for the management of acute migraine with aura [7]. However, evidence supporting the efficacy of cortical stimulation varies across different approaches, and recommendations for pain conditions other than chronic pain are inconclusive due to limited experience [8,9,10]. Among the emerging areas of stimulation, the cerebellum stands out as a potentially pivotal region. In recent years, growing evidence has highlighted the cerebellum’s involvement in pain processing [11,12,13]. Considering its wide connections with different cortical and subcortical areas, it has been proposed that the cerebellum integrates multiple neural processes, including sensorimotor control, affective processing, and pain modulation. Moreover, in recent years, much attention has been directed towards studying cerebellar activity and connectivity using conventional non-invasive brain stimulation techniques, primarily TMS and tDCS, as well as more advanced techniques, such as combining TMS and electroencephalography or magnetoencephalography (MEG) [14,15].

The purpose of the present review is to integrate current knowledge about the role of the cerebellum in pain perception with novel biomarkers of cerebellar activity and non-invasive brain stimulation techniques used to modify cerebellar excitability. Our aim is to provide an account of the recent advancements in these topics and to explore the potential of combining these techniques to elucidate the cerebellum’s role in pain control, offering perspectives for innovative therapeutic avenues in chronic pain management. We will also highlight some unresolved issues concerning the application of cerebellar stimulation in pain relief, discussing technical and pathophysiological aspects which need further assessment.

## 2. Role of the Cerebellum in Pain Processing

Traditionally, the cerebellum has been acknowledged as a central structure in movement physiology, and its role is preserved across species of sufficient size and motility, being influenced by inertia in their kinematics [16]. The role of the cerebellum as a dynamical state estimator for movement is well known, and its effect is exerted through multiple motor correction feedback loops. For example, climbing fibres from the inferior olivary nucleus project to the sensorimotor portions of the cerebellum, providing information to refine ongoing kinematic programs through their interaction with Purkinje cells [16]. The motor role of the cerebellum is further substantiated by two cortico-cerebellar loops, i.e., the cortico-ponto-cerebellar and the cerebello-thalamo-cortical loop [16]. While information conveyed by the former derives from sensorimotor regions such as the prefrontal, supplementary motor, premotor, and primary motor areas, the latter serves as an output pathway from deep cerebellar nuclei and provides immediate motor feedback originating principally from the anterior cerebellar lobe [17,18,19,20]. Inputs from the olivary nucleus and movement programs relayed by cortico-ponto-cerebellar pathways are integrated in the cerebellum, which then provides a corrected output that is conveyed through the cerebello-thalamo-cortical pathway, enabling smooth and precise movement. Nonetheless, the cerebellum is involved in more than just motor function tuning. For example, the cortico-ponto-cerebellar loop also contains projections from prefrontal, multimodal posterior, temporal, paralimbic, posterior para-hippocampal, and visual areas. These deal with numerous functions, ranging from planning and foresight for the prefrontal cortex to memory and visuo-spatial attention for parieto-temporal regions [16,21]. Cerebellar involvement in non-motor functions is confirmed by a cognitive–affective syndrome in patients affected by cerebellar lesions, also known as Schmahmann’s syndrome [22]. A cohort study found cognitive–affective deficits in 64% to 86% patients with cerebellar stroke with transient affective-behavioural and longer-standing depressive symptoms. In concordance with the distributed nature of the afferences of the cortico-ponto-cerebellar pathway, memory, central processing speed, and linguistic abilities were involved [23]. Crucially, the anterior insula, a central area involved in pain modulation and autonomic responses, is among the non-motor areas strongly connected with the cerebellum [18]. This supports the hypothesis that the cerebellum is connected to the pain and salience network [24]. Pain perception in humans is a complex process that relies on different levels of integration. Peripheral perception is mediated by unmyelinated C fibres, myelinated A-delta fibres, and, in pathologic conditions and for some types of pain (e.g., shock-like pain), A-beta fibres [25]. These fibres synapse in the posterior columns of the spinal cord after a brief ascent in the posterior gelatinous substance, then cross the midline anterior to the central canal, and ascend as the spinothalamic tract to the ventral posterior lateral nuclei of the thalamus. From here, information is distributed to the sensory cortices and anterior insula, where pain localization and discrimination occur [26,27]. A parallel spino-parabrachial pathway synapses onto the parabrachial nucleus in the brainstem, and from here, information is distributed across a network comprising the periaqueductal grey, hypothalamus, amygdala, insula, and cingular cortex to elaborate the affective and emotional components of pain [28]. These systems are joined by the trigemino-thalamic tract, collecting afferents from the facial district that are then distributed to the ventral posterior medial nuclei of the thalamus and by some fibres of the spinal lemniscus, collecting afferents from dorsal columns and conveying only shock-like pain information to the thalamus [29,30].

The role of the cerebellum in pain processing might be justified by its evolutionary role as a dynamic movement integrator, given that pain information can be crucial for movement organization and direction, especially in lower species; this is also supported in humans by the presence of areas in the posterior cerebellum that exhibit common responses to movement and pain processing, suggesting the functional integration of this information [16,31]. From a physiological point of view, A-delta and C fibres afferences can activate Purkinje cells, as shown in mammal studies [11,32]. Two different afferent sensory pathways have been suggested, a spino-olivo-cerebellar one, with afferences from A delta and C fibres reaching the anterior lobe ipsilateral to stimulation, and a spino-ponto-cerebellar one, which conveys C fibre inputs to the cerebellar vermis. Both are not fully characterized in humans and mammals, and their precise organization is still a matter of debate [33,34]. Another possible afferent pathway has been hypothesized based on indirect evidence from rat models, where spino-reticular tract neurons, projecting to the lateral reticular nucleus, a pre-cerebellar nucleus, were found to be responsive to noxious stimuli [35]. Further support for the involvement of the cerebellum in pain processing comes from the observation of altered gene expression in the cerebellum of rat models of chronic pain, which correlated with altered nociceptive sensitivity [36]. Pain information elaborated through the cerebellum is integrated into cortical networks through connections with the periaqueductal grey, sensory cortex, dorsolateral prefrontal cortex, basal ganglia, hippocampus, hypothalamus, and amygdala [16]. Human studies have confirmed the role of the cerebellum in pain processing. A meta-analysis of 47 neuroimaging studies found evidence for a response to pain in the vermis and posterior cerebellar hemisphere [11]. Moreover, the cerebellum is involved in pain gauging and expected pain elaboration, as evidenced by a functional magnetic resonance imaging (fMRI) study that found that pain-induced activation in lobule VI and the anterior vermis varied with subject reports of pain intensity, though only when stimuli were self-administered [37]. Similarly, a positron emission tomography (PET) study showed thermal pain intensity-dependent activity in the anterior cerebellum [38]. As further clinical support for this evidence, a lesional study involving 30 patients showed that thermal and pinprick pain sensitivity increases after cerebellar stroke, in parallel with a reduction in offset analgesia, with no variation in pain threshold [39].

## 3. Novel Biomarkers for Electrical Cerebellar Activity in Humans

Non-invasive investigations into the electrophysiology of the human cerebellum remain largely unexplored compared to explorations of the cerebral cortex. The non-invasive recording of cerebellar electrical activity is challenging due to several physical factors, including the larger distance between cerebellar cortex and the scalp compared to the cerebral cortex and the “closed field” geometry of Purkinje cells, which may reduce scalp electroencephalography (EEG) amplitudes below the detection threshold [40,41]. Several physiological factors may also contribute to the difficulties of the non-invasive recording of cerebellar electrical activity, such as a lower amplitude compared to the cerebral cortex, a lack of sufficient synchrony across cerebellar neurons, and the fact that cerebellar activity is tuned to high frequency (up to 200 Hz), with a correspondingly low signal-to-noise ratio at the scalp level [42,43]. For these reasons, investigations of cerebellar physiology have mostly been conducted using indirect methods. One example is eyeblink classical conditioning (EBCC), an associative learning protocol thought to rely on olivo-cerebellar circuits, in which a sound is conditioned to provoke an eyeblink in the absence of the unconditioned stimulus, the latter usually represented by an electric shock to the supraorbital nerve [44,45,46,47]. By applying TMS to one cerebellar hemisphere, it is possible to test the physiology of the cerebello-thalamo-cortical (CTC) pathway, either by testing the inhibitory effect of cerebellar stimulation on the contralateral M1 (a protocol named cerebellar brain inhibition, CBI) [48,49,50] or by recording cerebellar transcranial evoked potentials, i.e., EEG responses evoked by cerebellar stimulation on the contralateral hemisphere [51,52].

The possibility for the non-invasive recording of cerebellar activity in intact human brains has recently been supported by several studies, with most of them focussed on defining signals related to the control of vestibular function by the cerebellum. Electrical activity, recorded by surface electrodes placed on the scalp over the cerebellum in a procedure named a electrocerebellogram (ECeG), is thought to reflect cerebellar local field potentials (LFPs); in particular, ECeG activity in the very-high-gamma frequency range (160–250 Hz) may be generated by assemblies of Purkinje cells connected via inhibitory recurrent axonal collaterals [41,42]. Todd and coworkers [53] recorded meaningful ECeG results at a frequency between 80 and 320 Hz from electrodes placed below the inion and found that this activity was modulated by moving visual stimuli. This is in line with invasive studies showing that both non-Purkinje and Purkinje cells are strongly modulated by optokinetic stimulation, with the rate of simple spikes of Purkinje cells being dependent on the speed of motion [54].

Further support for the notion that the ECeG mostly stems from the vestibular portion of the cerebellum comes from the observation that it is modulated during vection with vestibular stimulation, which causes a reduction in the power of the ECeG and an increase in cerebro-cerebellar EEG coherence [55]. In addition to spontaneous activity, evidence exists for cerebellar responses recorded non-invasively by a number of stimuli activating the vestibular system. Vestibular evoked potentials have been recorded by electrodes over the posterior fossa in a number of studies employing visuo-vestibular stimulation [56] and classical eyeblink conditioning paradigms using mastoid taps, which are believed to activate otolith receptors [57] or auditory tones [58]. Cerebellar evoked responses have also been recorded following impulsive acceleration applied on the mastoid and the trunk [59,60], opening new possibilities to explore cerebellar involvement in postural control. This avenue has resulted in a small number of clinical applications so far, with two studies suggesting that a decrease in cerebellar theta activity is linked to postural instability and the freezing of gait in Parkinson’s disease [61,62].

Data about the possibility to record electrical cerebellar activity related to the control of upper limbs are more limited. Todd and colleagues [63] found significant cerebellar activity related to ipsilateral ballistic movements of the finger. Pan and colleagues [64] observed increased cerebellar oscillatory EEG power in patients affected by essential tremor, mostly involving the upper limbs, in a frequency range compatible with tremor activity. Increased ECeG power in a broad range of frequencies was found in a subsequent study on essential tremor [65], and this abnormal activity was suggested to be correlated with tremor severity in familial cases.

## 4. Non-Invasive Brain Stimulation Techniques Used to Modulate Cerebellar Activity in Humans

Non-invasive brain stimulation (NIBS) offers safe and well-tolerated means to modulate neural activity without invasive interventions [66,67,68]. These methods target specific cortical regions or nodes within neural networks, thereby altering their associated functions [69], facilitating a deeper understanding of neural dynamics and paving the way for innovative therapeutic interventions and neuroscientific inquiries. Here, we give a brief account of the main NIBS techniques and their effects when used to modulate cerebellar activity.

### 4.1. Transcranial Magnetic Stimulation

TMS is a non-invasive neurostimulation technique that involves the application of magnetic pulses to specific areas of the brain [70]. TMS operates on Faraday’s principle of electromagnetic induction to generate electrical currents. The rapid change in the magnetic field produced by TMS induces a secondary current in nearby conductors, including the brain, when it is applied over the scalp. These induced currents can either excite or inhibit neuronal activity, depending on the parameters of stimulation [71]. Over the nearly four decades since its inception, TMS has served as a valuable tool for examining intracortical, cortico-cortical, and cortico-subcortical interactions [71,72,73].

#### 4.1.1. Repetitive TMS (rTMS)

RTMS involves the repeated delivery of magnetic pulses at a specific frequency, with variable inter-stimulus intervals. The protocols most commonly used are divided between high-frequency (>5 Hz) and low-frequency rTMS (≤1 Hz), which lead to increased and decreased neuronal excitability, respectively, mediated by long-term potentiation (LTP)- and long-term depression (LTD)-like mechanisms. These effects have been tested widely on the cerebellum, where rTMS has been applied to increase or decrease cerebellar output to M1 or to study the effects of cerebellar stimulation on motor learning, coordination, and cognitive functions [74,75,76]. For instance, 1 Hz cerebellar rTMS results in increased MEP amplitude and decreased intracortical facilitation (ICF), which reflect a reduction in the cerebellar inhibitory control over M1 [77,78,79]. This inhibitory effect has also been confirmed in behavioural studies, as Torriero and colleagues reported reduced procedural learning after 1 Hz cerebellar rTMS [80]. Although rTMS can be easily used to induce excitability changes in the cerebellum, it has some practical issues, such as the prolonged stimulation time, which can result in patient discomfort and coil overheating.

#### 4.1.2. Theta Burst Stimulation (TBS)

Theta Burst Stimulation (TBS) is a TMS protocol that has been proposed in more recent years in order to overcome some practical issues of rTMS, such as the prolonged stimulation time. TBS is a form of rTMS that utilizes short high-frequency trains (bursts) at a predefined repetition rate in order to induce long-lasting focal changes in cortical or cerebellar excitability [81,82]. Similar to standard rTMS, both excitatory and inhibitory effects can be induced, depending on whether TBS is delivered intermittently (iTBS) or continuously (cTBS). ITBS consists of three pulses at 50 Hz, repeated at 5 Hz intervals, applied in 2 s trains repeated every 10 s, for a total of 190 s, which induces LTP-like facilitation, while cTBS involves the same bursts applied continuously for 40 s and generally induces LTD-like inhibitory effects [83,84]. The overall 600 TBS pulses are sufficient for inducing physiologic effects that last >1 h (which is longer than the traditional 1 to 20 Hz rTMS protocols), with the advantage of being applied in a very short period of time. Several studies have demonstrated that TBS protocols are able to induce bidirectional and long-lasting changes in the excitability of the cerebello-thalamo-cortical circuits in humans and, therefore, able to activate different mechanisms of synaptic plasticity when applied over the cerebellum [85,86]. Similar results were obtained by Popa and coworkers, who assessed the impact of iTBS and cTBS on different cortico-cortical measures, showing distinct after-effects. For instance, cTBS reduced short intracortical inhibition (SICI) and CBI and decreased long intracortical inhibition (LICI) and MEPs, whereas iTBS increased LICI and MEPs [87]. Moreover, Halko and colleagues observed a significant modulation of the cerebral default mode network (DMN) after iTBS of the lateral cerebellar Crus I/II and of the cerebral dorsal attentional network after stimulating the vermal lobule VII with iTBS [88]. Conversely, Farzan and coworkers showed that cerebellar iTBS increased the complexity of brain signals in a network-specific manner and observed a region-specific shift in the power of cortical oscillations toward higher frequencies [89].

#### 4.1.3. Paired Associative Stimulation (PAS)

In its original form, paired associative stimulation (PAS) consists of repetitive, low-frequency peripheral nerve stimulation combined with TMS over the contralateral motor cortex, with the two being separated by an ISI appropriate for inducing spike timing-dependent plasticity (STDP), a form of plasticity based on the Hebbian rule [90,91,92]. In modified versions of PAS, peripheral stimulation can be replaced by cortical or cerebellar TMS [93,94] or visual stimuli [95,96]. In a study by Lu and coworkers, the authors applied a cerebellar conditioning stimulus over the inion using a double-cone coil, followed by a target stimulus over the left M1 hand area with a figure-of-eight coil at ISIs of 2, 6, and 10 ms. They observed MEP potentiation with an ISI of 2 ms, while ISIs of 6 and 10 ms resulted in MEP depression lasting 30–60 min after PAS. Interestingly, the protocol did not affect ICF but significantly reduced CBI and SICI [94], suggesting a non-specific effect on inhibitory circuits. In another study by Pauly and colleagues, cerebellar PAS using two TMS pulses—one over the target area VIIIA, followed by the second over M1—decreased MEP amplitude [79].

### 4.2. Transcranial Electrical Stimulation (tES)

Transcranial electrical stimulation (tES) consists of a low-intensity current delivered through two or more electrodes placed on the scalp, serving as cathodes or anodes. Their size varies, and for cerebellum tES, it is normally 5 cm × 5 cm [97]. In this case, the active electrode is positioned directly over the cerebellum, while the return electrode is placed either over the buccinator muscle or the right shoulder. The stimulating electrode may be placed over one or both cerebellar hemispheres, typically positioned 1–2 cm below and 3–4 cm lateral to the inion. Alternatively, it can be centred on the median line 1–2 cm below the inion [98]. A crucial technical consideration in cerebellar tES is the placement of the return electrode and the orientation of the derived current flow and electrical field. Consequently, the specific position chosen for the return electrode plays a significant role in determining the nature and extent of the elicited changes [99]. Here, we will discuss the most common forms of cerebellar tES used in the literature: anodal or cathodal tDCS, transcranial alternate current stimulation (tACS), and transcranial random noise stimulation (tRNS).

#### 4.2.1. Transcranial Direct Current Stimulation (tDCS)

TDCS involves the administration of a weak (1–2 mA) direct current, between two or more electrodes, usually for 15–25 min, causing alterations in the neuronal resting membrane potential, with polarity-specific effects: anodal stimulation generally enhances cortical excitability, while cathodal stimulation is thought to operate in an opposite way by depolarizing or hyperpolarizing the membrane potential [100]. These effects on membrane polarization are proposed to be paralleled by changes in spontaneous firing rates [101,102]. Cerebellar tDCS can modulate the membrane polarization of Purkinje and glial cells, as well as mossy and climbing fibres [103,104]. However, the consistency and reproducibility of these effects are limited due to the significant variability among participants; this may depend on several factors, including neuron orientation, and may suggest that the general rule of anodal being excitatory and cathodal inhibitory is probably an oversimplification of the physiological mechanisms underlying tDCS [105]. Computational modelling studies have suggested that weak exogenous electric currents at an intensity of 2 mA can penetrate the outer layers of the cerebellar cortex. Experimental research findings have demonstrated that cerebellar tDCS can elicit neurophysiological alterations in cerebellar–brain interactions [86] and has the potential to impact gait adaptation, motor learning, and cognition in healthy individuals [106,107,108,109]. For instance, cerebellar tDCS has been found to interfere with motor cortex synaptic plasticity during PAS involving the median nerve and motor cortex, suggesting the cerebellum’s role in synchronizing sensory input and motor output [110]. In another study, Galea and colleagues found that cathodal tDCS resulted in a decrease in CBI, whereas anodal tDCS increased it. These polarity-dependent effects align with the notion of respective decreases and increases in the Purkinje cell-mediated inhibition of M1 [111].

#### 4.2.2. Transcranial Alternating Current Stimulation (tACS)

Differently from tDCS, tACS consists of the application of a weak (1–2 mA) alternate current that is thought to entrain cortical oscillations by inducing coherent changes in neuronal firing patterns and timing [97,112], a phenomenon recognized as ‘resonance principle’ [113,114]. To date, tACS has been widely applied in both healthy individuals and neurological patients, targeting diverse cortical regions, including M1, DLPFC, and the parietal and visual cortex [114,115,116,117,118,119,120,121]. An in vivo animal study demonstrated the ability of tACS to modulate the spiking activity of Purkinje cells and entrain them across a broad spectrum of frequencies [122]. In line with animal studies indicating that cerebellar neurons show intrinsic oscillatory properties at theta and gamma frequency bands with a functional role in motor control [123,124], cerebellar tACS delivered at 5 Hz (theta) and 50 Hz (gamma) modulates human motor behaviour [50,117,125,126,127]. Particularly, cerebellar theta-tACS decreases movement regularity during rhythmic finger tapping and increases the duration of a reaching task in healthy subjects [128]. Lastly, cerebellar gamma-tACS improves the acceleration of voluntary movements during a rapid learning task in healthy subjects [129].

#### 4.2.3. Transcranial Random Noise Stimulation (tRNS)

In TRNS, a current randomly fluctuating across a wide range of frequencies, typically between 0.1 Hz and 640 Hz, is delivered [130]. These random fluctuations are thought to increase cortical excitability by promoting stochastic resonance, a phenomenon in which weak signals become amplified in the presence of noise [131]. The effect of tRNS is thought to involve depolarization of the cell membrane by the activation of sodium channels [132]. Only one study by Kawamaki and colleagues has explored cerebellar-cortical pathways with the purpose of comparing the effects of tRNS and tDCS over the cerebellum using CBI as an indicator of cerebellar excitability. The authors reported a significant correlation between CBI and MEPs following tRNS, with a decrease in CBI accompanied by an increase in contralateral MEPs amplitude [110].

## 5. Cerebellar Neurostimulation Studies Addressing Pain Perception in Humans

As previously described, exploring cerebellar activity could offer a novel and intriguing approach for investigating the pathophysiology of pain perception. Additionally, there is increasing evidence indicating the cerebellum as a focal point for therapeutic interventions.

Table 1 summarizes studies on cerebellar NIBS interventions for managing chronic pain.

Zunhammer and coworkers first used NIBS to study the involvement of the cerebellum in pain processing [133]. In their study, 1 Hz rTMS was delivered over lobule VII of the cerebellar vermis and over Crus II of the right lateral cerebellar hemisphere. Cold and warm detection thresholds were used as a surrogate for changes in nociception. The authors found that rTMS over the lateral cerebellum is able to increase the heat threshold. Interestingly, the same result was found when rTMS was delivered over the neck as a control condition; therefore, assessing whether the effects arise from the activation of peripheral afferents or cerebellar pathways provides demanding challenges [133]. Subsequently, Bocci and colleagues assessed the role of the cerebellum in pain perception using tDCS [12]. In their study, fifteen healthy volunteers underwent 2 mA anodal cerebellar tDCS (atDCS) and cathodal cerebellar tDCS (ctDCS). Laser-evoked potentials (LEPs) and a Visual Analog Scale (VAS) for pain were used as behavioural and neurophysiological readouts. More precisely, the N1 and N2/P2 LEPs components were studied. The results showed that ctDCS increases pain perception, as well as the amplitude of LEPs, and decreases the latencies of the latter. Conversely, atDCS induced opposite effects. As modulation was observed in both N1 and N2/P2 components, the authors proposed that cerebellar involvement may extend to modulating both somatosensory and cingulate cortices. Following these findings, the same research group endeavoured to explore the potential of tDCS as a therapeutic intervention for phantom limb pain [134]. Fourteen amputee patients were recruited and divided into two groups, one receiving sham stimulation and another receiving atDCS for 5 consecutive days. The authors used both clinical and LEPs outcomes. Cerebellar atDCS was able to improve paroxysmal pain and non-painful phantom limb sensation; moreover, it reduced N1 and N2/P2 amplitudes, confirming findings reported by the same group [12].

In another study, cerebellar stimulation was also used to ameliorate lower-extremity pain symptoms [13]. In this study, anodal, cathodal, and sham tDCS were delivered. The real stimulation protocol involved administering five minutes of 2 mA tDCS, with the active electrodes positioned 2 cm below the inion. The lateral borders of the electrodes were situated approximately 1 cm medial to the right mastoid apophysis, while the reference electrode was placed over the buccinator muscle. Pain threshold was used as a readout. The results showed that anodal tDCS increases lower-extremity pain threshold compared to sham and cathodal stimulation. This study has some differences compared to the work of Bocci and colleagues [12]. First, the tDCS montage was different: Bocci and colleagues placed the reference electrode over the shoulder, while Pereira and coworkers used a cephalic montage (reference over buccinator muscle). Moreover, Pereira and colleagues did not use any neurophysiological measures to evaluate the effect of electrical stimulation. Recently, Stacheneder and coworkers used tDCS to assess the role of the cerebellum in nociception and endogenous pain modulation [135]. In this work, anodal and cathodal tDCS was delivered using an extracephalic montage with the reference electrode placed over the lateral upper arm. Readouts included the RIII reflex, offset analgesia (OA), conditioned pain modulation (CPM), somatosensory evoked potentials, and subjective pain ratings. The results showed that cathodal tDCS reduced pain thresholds, increased N120 amplitude, and increased RIII reflex area. On the other end, anodal tDCS increased OA. The authors concluded that cathodal tDCS increased pain perception and reduced endogenous pain inhibition, while anodal tDCS increased endogenous pain inhibition [135].

It is important to note that, despite the promising results, the mentioned studies suffer from some limitations that restrict the generalizability of their findings. These limitations include limited sample sizes, as well as variability in key parameters such as stimulating electrode placement, current intensity, and methods for pain assessment. Another important point is that the effects of stimulation were monitored for a very short time; therefore, inferences on the extent of possible long-term clinical benefits are limited.

## 6. Conclusions and Future Directions

**Figure 1:**
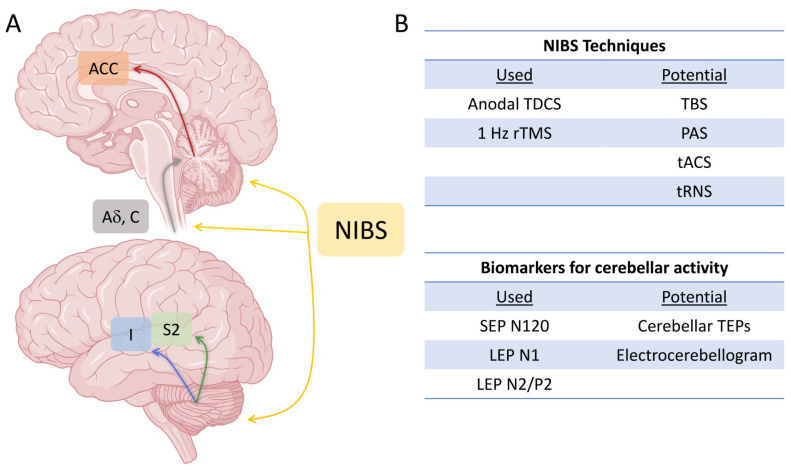
Schematic representation of possible mechanisms of action of cerebellar NIBS in the con text of pain modulation (**A**) and list of NIBS techniques and biomarkers for cerebellar activity used that have been in previous studies or are of potential interest (**B**). See text for details. ACC: anterior cingulate cortex; I: insular cortex; S2: secondary somatosensory cortex.

In recent years, the cerebellum’s significance in non-motor functions, including pain perception, has garnered recognition. This acknowledgment coincides with a notable surge in NIBS methodologies capable of modulating the excitability of cerebellar circuitry. Additionally, various techniques for assessing cerebellar function, both directly and indirectly, have emerged. These advancements, however, have not been paralleled by a similar increase in the exploration of cerebellar NIBS applications for managing chronic pain in human subjects. Figure 1 summarizes the possible mechanisms of action of cerebellar NIBS in pain control and outcome measures which have been employed or may be of potential use to assess the effects of cerebellar NIBS on pain. Existing works primarily report positive outcomes of anodal cerebellar tDCS [12,13,134,135] and 1 Hz rTMS [133]. The underpinning of pain modulation by these techniques has still not been clarified. Given the report that a decrease in pain was achieved when 1 Hz rTMS was applied over the neck [133], a placebo effect, or an effect on peripheral afferents, in particular A-delta and C fibres to the cerebellum [11,32,33,34], cannot be excluded. Another possibility is a direct action on the cerebellum [36]. While electrical field modelling suggests the specificity of unilateral cerebellar tDCS for the posterior aspect of the targeted hemisphere [136], little is known about the neuronal basis of tDCS after-effects. tDCS applied to brain slices in mouse models has been shown to induce LTP, mediated by brain-derived neurotrophic factor and Tyrosine kinase B activation [137]. The modulation of neurotransmitters involved in the regulation of synaptic plasticity, such as GABA and glutamate, has been observed after cerebellar tDCS [138]. The effects of tDCS might also be mediated by changes in the activity of Golgi cells, including prolonged spiking after membrane depolarization lasting for minutes [139]. The effects of cerebellar rTMS might be akin to those induced by tDCS, as suggested by the similar effect of the two techniques on PAS and CBI [111,140,141,142]. This possibly suggests the presence of cerebellar neurons with similar sensitivity to tDCS- and rTMS-induced modulation [67]. However, the mentioned mechanisms may not be specific for the effects of cerebellar NIBS on pain, and further studies are needed in this regard.

It is possible that the effects of cerebellar NIBS are mediated by effects on areas of the pain network, such as the secondary somatosensory area, the insular cortex, and the anterior cingulate cortex [24]. This latter hypothesis is supported by changes in electrophysiological markers of activity in these areas, including the N120 component of SEPs [135,143,144], as well as the N1 and N2/P2 components of LEPs [12,134].

There are important areas for improvement regarding the applications of cerebellar NIBS in pain control. Key elements are the standardization of conditioning parameters and improvement of sham stimulation, which may prove difficult in most cases [145,146,147]. This is a crucial point, considering the sizeable placebo effect often occurring in pain studies [148]. Further, no formal study on the safety and tolerability of cerebellar NIBS has been carried out; this limits its applicability in the clinical setting and evaluations by regulatory entities such as the FDA or EMA. Nonetheless, in consideration of promising initial results, serious investment toward larger and rigorous randomized controlled trials and safety would be advisable to allow for implementation in the clinical setting.

In conclusion, our review underscores the untapped potential of further investigations that integrate cerebellar NIBS with non-invasive assessments of cerebellar function in the context of chronic pain treatment. We hope that our work will prompt further research in this domain.

## Figures and Tables

**Table 1:** Features of studies investigating the use of NIBS for chronic pain in humans. atDCS, anodal cerebellar tDCS; ctDCS, cathodal cerebellar tDCS; CP, chronic pain; rTMS, repetitive transcranial magnetic stimulation; RMT, resting motor threshold; CT, cold detection threshold; HPT, heat pain threshold; LEPs, laser-evoked potentials; UL, upper limb; CPM, conditioned pain modulation; SEPs, somatosensory evoked potentials.

Authors	Sample	NIBS Technique and Outcome Measures	Stimulation Areas, Electrode Montage, or Direction of the Stimulation Coil	Behavioural and/or Neurophysiological Outcomes
Zunhammer et al. (2011) [133]	10 CP patients	1 and 10 Hz rTMS at 120% RMT and 1000 stimuli compared to sham or neck magnetic stimulation	Medial→Lobule VII of the cerebellar vermis; lateral→Crus II of the right lateral cerebellar hemisphere Coil handle pointing upwards	1 Hz rTMS over lateral cerebellum and neck significantly increased HPT and decreased CT
Bocci et al. (2015) [12]	15 CP patients	20 min of anodal, cathodal, or sham cerebellar tDCS at 2 mA; 10 LEPs at 3 timepoints (before, immediately after and 60 min after stimulation) and changes of RMT using TMS (before, after stim and after 60 min)	Bilateral cerebellar hemispheres ctDCS→cathode 2 cm below inion, anode on ipsilateral shoulder atDCS→anode 2 cm below inion, cathode on ipsilateral shoulder	ctDCS increased pain perception, as well as the amplitude of N1 and N2/P2 LEPs, and decreases the latencies of the latter, while atDCS induced opposite effects
Bocci et al. (2019) [134]	14 unilateral UL amputees	20 min and 5-day sessions of sham and anodal cerebellar tDCS at 2 mA; LEPs at at 3 timepoints (before, immediately, and 2 weeks and 4 weeks after stimulation)	Bilateral cerebellar hemispheres; ctDCS→cathode 2 cm below inion; anode on ipsilateral shoulder atDCS→cathode 2 cm below inion; anode on ipsilateral shoulder	Cerebellar atDCS improved paroxysmal pain and non-painful phantom limb sensation; it reduced N1 and N2/P2 amplitudes
Pereira et al. (2017) [13]	14 CP patients	5 min anodal, cathodal, or sham cerebellar tDCS at 2 mA	atDCS→anode 2 cm below inion; cathode on ipsilateral buccinator muscle ctDCS→cathode, 2 cm below inion; anode on ipsilateral buccinator muscle	atDCS increased low-extremity pain threshold compared to sham and cathodal stimulation
Stacheneder et al. (2023) [135]	21 CP patients	20 min anodal, cathodal, or sham cerebellar tDCS at 2 mA, RIII reflex, offset analgesia, CPM effect and SEPs recorded 0, 30, and 60 min after stimulation	ctDCS→cathode 2 cm below inion; anode over the shoulder atDCS→anode 2 cm below inion; cathode over the shoulder	ctDCS reduced pain thresholds, increased N120 amplitude, and increased RIII reflex area, while atDCS increased OA

## Data Availability

No new data were created or analysed in this study. Data sharing is not applicable to this article.
